# The role of customers’ awareness towards the sustainable development goals (SDGs) of banks on their behavior

**DOI:** 10.1007/s11356-022-23111-8

**Published:** 2022-09-22

**Authors:** Athanasia Stauropoulou, Eleni Sardianou, George Malindretos, Konstantinos Evangelinos, Ioannis Nikolaou

**Affiliations:** 1 Department of Economics and Sustainable Development, Harokopio University, El. Venizelou 70, 17671 Athens, Greece; 2 Centre for Environmental Policy and Strategic Environmental Management, Department of Environment, University of the Aegean, Mytilene, 81100 Lesvos Island, Greece; 3 Business and Environmental Technology Economics Lab, Department of Environmental Engineering, Democritus University of Thrace, 67100 Xanthi, Greece

**Keywords:** Customer awareness, Banking customers, Sustainability practices, Customer trust, Loyalty and behavior, SDGs

## Abstract

Banks and sustainable development have lately gone hand in hand. Of late, banks have focused on sustainable management in order to improve their environmental footprint, to eliminate financial risks, to promote social issues, and to exploit new opportunities. The sustainable development management of organizations attracts new customers over and above conventional institutions thus leading to greater market share and increased revenues. This paper aims to examine if and how sustainable development goals (SDGs) adopted by banking institutions play a role in customers’ decisions and behavior. The way that banks, SDGs, and sustainable image affect customer behavior, attitudes, trust, loyalty, satisfaction, and perceived fair pricing policy is assessed. To this end, 1084 questionnaires were collected and the PLS-SEM method was utilized. The findings show a positive relationship between the stance of banks relating to SDGs and customer trust, loyalty, and perceived fair pricing policy. Finally, the SDGs adopted by banks are an important strategic tool which strengthens relationship with their customers.

## Introduction

Banks operate under intense competition and a demanding customer base. Banks strive for their viability by undertaking measures to be profitable and increase their market share. Corporate environmental management and sustainability are fields where organizations and business, including banks, can differentiate in the market and increase their competitiveness. However, banks adopt sustainability management practices in a variety of different ways.

Firstly, sustainability management in the banking sector is adopted as a way to protect debtors’ and lenders’ investments from likely environmental and social risks which could be transferred to banks mainly through the inability to pay the loan installments (McKenzie and Wolfe 2004). Such environmental risks of debtors and lenders could result in significant financial losses due to natural disasters (e.g., because of extreme weather events) to restore physical damages and could result in heavy penalties for non-compliance with environmental regulations. Secondly, banks view sustainability issues as an opportunity to make new products for businesses and households (e.g., energy saving buildings, environmental investments of firms, green consumer cards) (Bătae et al. 2021). Thirdly, financial institutions adopt sustainability management to decrease their environmental footprint and improve their image through sustainability management practices (Adu-Gyamfi et al. 2022). One perennial question in the field of corporate sustainability, which is also significant in the banking sector, is why organizations adopt sustainability management practices (including SDGs). Taking a general overview of corporate environmental management, various theories have been examined such as the stakeholder theory, the institutional theory, the legitimacy theory, the resource-based theory, and the knowledge-based theory (Delmas and Toffel 2004; Campbell 2007). The merit of each theory depends on the sector, the economic background, and institutional context in which an organization operates. Regarding the question of why banking institutions adopt sustainability management, some explanations given are the reduction of potential financial risks from non-compliance with environmental regulations and improvement of their social and environmental image to attract socially responsible customers. Naturally, like any other organization, banks include social and environmental dimensions in their business strategy to attract customers who seek sustainable responsible financial products. The integration of sustainability management into the strategic planning of banks may have the effect of reinforcing their core objectives and simultaneously, minimize their environmental footprint. Concurrently, a higher level of environmental and social awareness is associated with a higher degree of transparency of processes, moral commitment, and ethical standards. Within this context, the issues that may arise related to moral hazard and unfavorable selection are mitigated (Gangi et al. 2019; Goss and Roberts 2011).

The sustainability strategy of banks is associated with the main goal of profit maximization. This is in line with the principal goal of every organization to increase their market share and customers who are increasingly becoming more environmentally sensitive. CSR and sustainability issues affect consumers’ preferences regarding products and services (Sardianou et al. 2017; Fatma and Rahman 2016; Pérez and del Bosque 2015a).

There is considerable literature examining the first two cases of banks and sustainability, while a more limited range focuses on banks as good sustainability performance organizations. By focusing on the last case, it is identified that current studies focus on examining sustainability performance of banking institutions towards triple-bottom-line and lately sustainable development goals (SDGs). Many studies have focused on examining how banks promote SDGs overall (Scheyvens et al. 2016; Suchodolski et al. 2022) and some specific SDG goals such as SDG 10 (Úbeda et al. 2022). Specially, the SDGs include a set of 17 goals suitable for promoting successful solutions for various economic, social, and environmental aspects. SDGs were launched by the United Nations through the 2030 Agenda in order to support economic prosperity, environmental protection, and social welfare issues in global and national contexts (UN Global Compact 2015). SDGs have been transferred into many sectors (including banks) (Bose and Khan 2022).

However, very little work has been carried out examining how the sustainability management of banks affects customer preferences; in other words, what is the relationship between the SDGs of banks and customer behavior. This paper aims to examine this relationship by investigating the influence of the banking sector’s sustainability management on customer intentions and behaviors vis-à-vis financial products. Particularly, how the sustainability performance of banks on SDGs affects their customers’ trust, loyalty, and satisfaction is examined. To answer such research questions, a questionnaire-based survey was conducted and a structural equation model developed. A total of 1084 questionnaires were collected from bank customers. The findings show that there is a positive relationship between banking sustainability (SDGs) performance and customer trust, loyalty, image, and perceived fair pricing policy. The findings are useful to policymakers and bank senior management, not only for incorporating sustainable practices in their strategy but also to disclosing these to their stakeholders.

The rest of the paper is divided into four sections. The first section describes the theoretical background regarding banks and sustainable development. Specifically, research hypotheses from a literature review were developed. The second section develops the methodological framework on which this study is based. The third section analyzes the findings of this paper and the last section outlines the conclusion and discussion.

## Theoretical underpin—research hypotheses

### Sustainable banking

Today, banking institutions are considered directly and indirectly responsible for achieving SDGs (EBF 2021). The new institutional framework creates suitable conditions in which banks should undertake specific compulsory and proactive practices to promote sustainability goals. A number of European Union regulations (EU regulation 2019/2088, EU taxonomy) and voluntary initiatives (Equator principles) encourage financial institutions to adopt sustainability and socially responsibility management practices. The Principles for Responsible Banking (alignment, impact and target setting, clients and customers, stakeholders, governance and culture, transparency and accountability) contribute to the positive involvement of the banking system to the social interest. This happens when banks adopt a sustainable voluntary banking framework and provide a socially responsible behavior. More precisely, by participating in the principles, banks from around the world they choose to become more responsible, supported by a global framework that aligns their business decisions with the broader goals of society (PRB 2019). Furthermore, Global Alliance for Banking on Values is an international network in which banks from all over the world participate with the aim of the banking system supporting transitions in terms of sustainability, i.e., integrating economic, social, and environmental changes (GABV 2009).

Many banks have adopted sustainability on two levels: firstly, in the provision of services (e.g., products and lending) and secondly in organizational operations. The first level entails the introduction of sustainability criteria into lending procedures and the creation of new financial products to promote environmentally friendly investments by firms. A number of methodologies (e.g., ESG indexes, SDGs) have been recommended to estimate corporate environmental and social risks in order to eliminate the risk to banks from lending to environmental risky firms (Griffiths 2018; Eliwa et al. 2021). Furthermore, many banks perceive the opportunity to create new financial services and products for firms and households to promote SDGs by encouraging production and consumption on green building, certification with eco-labels (e.g., ISO 14001, EU eco-flowers label), green products and services (Sachs et al. 2019; Wellalage and Kumar 2021; Kumar and Prakash 2020). Also, financial institutions in collaboration with stakeholders such as regulators, investor, policymakers, and companies can improve implementation on environmental, social, and corporate governance issues, through partnerships, investments, and financing that promotes sustainable development (SSE initiative). The second level includes the adoption of sustainability practices (e.g., SDGs) from banks mainly to improve their sustainability performance as organizations. To achieve this goal, many banks adopt practices to improve their sustainability performance such as EMAS, energy management, waste management, CSR strategies, and sustainability reporting strategies (Pérez and Rodríguez del Bosque 2012; Pérez et al. 2013). Many studies have identified a positive relationship between sustainability practices, sustainability disclosures, and banking sector financial performance (Aras et al. 2018; Nizam et al. 2019). Similarly, some studies seek to examine the way that these sustainability practices of banks affect consumers (Poolthong and Mandhachitara 2009).

### Hypotheses development

Similar to other organizations, banks seek to improve their image in order to satisfy current customers and attract new ones to increase their market share and revenue. After the subprime mortgage crisis in 2008, the banking sector ethics was called into question as it was held responsible for the ensuing financial crisis. As seen in the previous section, however, the responsibilities of the banking system were not limited to financial matters but also extended to social and environmental issues (Paulet et al. 2015). Environmental and social impacts are identified either directly as organizations that destroy the natural environment or indirectly by financing business projects and consumption patterns of households that damage the environment through their actions. In general, sustainability management is considered as “good practice” for organizations in order to build an ethical image.

The demand for more environmental and social responsibility has increased the pressure on banks to develop sustainability strategies and their accountability to stakeholders. One of the strategies adopted is the 17 SDGs.

**Fig. 1:**
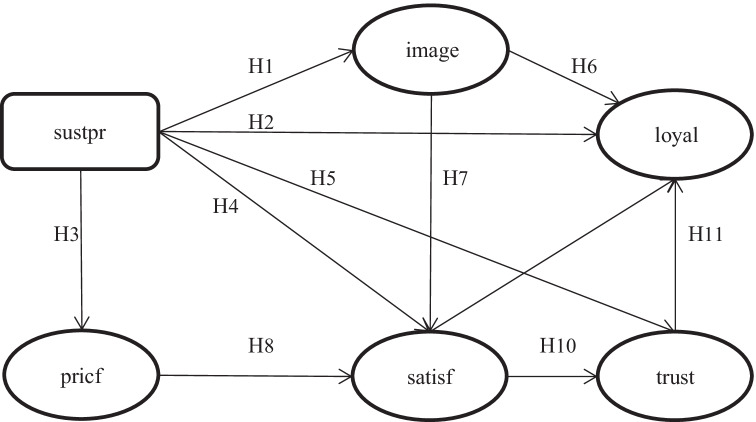
Research model

Figure 1 presents the research model of our analysis and the research hypotheses developed. Given that the aim of the study to is to explain the influence of awareness towards sustainable banking practices to consumers’ behavior, we develop a holistic research model that takes into account all dimensions of customers’ behavior. For this purpose, as we explain below, we examine the effect of sustainability awareness on customers’ image (H1), loyalty (H2), fair pricing (H3), satisfaction (H4), and trust (H5). In addition, we test the interrelation of the previously mentioned dimensions of customers’ behavior towards awareness regarding banking sustainability by developing H6–11. All these research hypotheses are analyzed by employing PLS-SEM analysis.

First, sustainability management practices have significant effects on the image of organizations. For example, organizations with a social orientation can gain an advantage which affect potential customer behavior. However, communication of sustainable responsible practices plays a critical role in reducing asymmetric information between organizations and customers about their progress in sustainability issues. Whereat, awareness of sustainable practices and related issues is speedily increasing in all sectors (Goyal and Chanda 2017). Increased communication and accountability promote stakeholder knowledge and awareness of sustainability-related activities and this enables them to reward responsible organizations (Sen and Bhattacharya 2001) while offering benefits to organizations from sustainability practices through better reputation (Servaes and Tamayo 2013).

It seems that the relationship between the choice of banks by depositors and its environmental performance does not follow a specific and stable pattern. Sometimes, this relationship seems to be negative (Galletta et al. 2021) and sometimes positive (Taneja and Ali 2021). However, research on the relationship between the 17 SDGs and customers’ decisions on whether to use a bank is inadequate. The 17 SDGs is expected to improve the profile of banks which are routinely criticized, especially during economic hardship, financial crises (the Lehman Brother economic crisis 2008), pandemics (COVID-19), and energy challenges (the Russian-Ukrainian war). Consequently, the integration of such practices and protection against potential risks will logically have a positive impact on customers.

The above analysis concludes with the following hypothesis:

- **Hypothesis 1 (H**_**1**_**):** The awareness of customers regarding the SDG practices of banks has a positive effect on their perceived image.

The sustainable management of banks could also affect the loyalty of their customers. In the field of corporate environmental management, organizations are encouraged to take into account customer perceptions of sustainable development, mainly in order to understand when changes in customer behavior and attitudes occur (Kuchinka et al. 2018). Similarly in the field of sustainable banking management, a positive relationship between sustainable banking management practices and customer loyalty seem to exist.

In this context, Pérez and Del Bosque (2015b) identified that the good CSR image of banks could affect customer behavior. Similarly, Ahmad et al. (2021a) have identified a positive relationship between different aspects of sustainability (e.g., economic, environmental, and social) and customer loyalty. Increased customer loyalty due to the sustainable management of banking institutions is also achieved with information exchange of customers through social networks. Through a questionnaire-based survey of 448 participants (289 male and 159 female) from bank customers, Ahmad et al. (2021b) identified that communicating the sustainability management practices of banks via social media has a positive impact on customer loyalty. Mandhachitara and Poolthong (2011) have identified a strong positive relationship between corporate social responsibility practices in the banking sector and customer loyalty.

Based on the above analysis, the following research hypothesis is proposed:

- **Hypothesis 2 (H**_**2**_**):** The awareness of bank customers about the SDG practices of banks has a positive effect on their loyalty.

Another significant feature that plays a critical role on bank customers is the pricing policy of banks. In order to evaluate the fair pricing policy, consumers must know the prices, profits, and costs. They usually tend to attribute the differentiation to the prices offered by competing firms, to achieving the desired profit rather than to cost. But when they are able to have some level of knowledge regarding the costing of the product, then this feeling is likely to be mitigated. In general, consumers seem to justify differences in prices and judge them as fair when such differences exist or are seen or justified in the quality of the products (Bolton et al. 2003). In other words, a comparative evaluation, conscious or unconscious, is needed to categorize the differentiation in the prices of competing companies. In this way, the fair or unfair tariff separation is also done policy involving two different structures of emotional response. So the identification of the elements that have given and characterized the pricing policy of a company as unfair can act as an auxiliary tool for the development of a correct strategy regarding the management of the pricing policy (Campbell, 1999). Fair pricing is a priority for the banking sector since the financial crisis of 2008 saw significant failures in the free operation of the financial markets (Martín-Consuegra et al. 2007). Today, corporate social responsibility and sustainability practices of banks greatly influence customers when they choose their bank and increase customers’ confidence in the financial products of banks. Matute-Vallejo et al. (2011) identified that improving CSR image in the banking sector makes customers less sensitive to financial costs and also shows that their perceptions about CSR and fair pricing influence their attitudes and behaviors in relation to banks.

The following research hypothesis is examined:

- **Hypothesis 3 (H**_**3**_**):** The awareness of bank customers regarding SDGs has a positive effect on their perceived fair pricing policy.

Customer satisfaction is a key factor for the banking sector; it helps create long-term bonds between customers and the organization. Some of the most significant factors examined relating to customer satisfaction are staff service, brand name, credibility, and competitiveness (Singh and Kaur 2011). In the new economic environment as it is after the 2008 economic crisis, Ruiz et al. (2014) pointed out the reliability and the leadership of management are two key factors that affect consumer satisfaction and trust in banks. Socially responsible behavior in banks and practices of sustainable management are steadily increasing. By examining 417 customers of 24 Jordanian banks, Srouji et al. (2019) identified a positive relationship between CSR practices and customer satisfaction. Similarly, Gunesh and Geraldine (2015) identified that philanthropic and CSR practices of banks create competitive advantage and contribute to customer satisfaction.

Green banking has also created conditions for security, trust, and convenience as well as value and a good environmental and social profile in the sector which affect customer satisfaction (Herath and Herath 2019). A questionnaire-based survey of 130 Taiwanese bank customers examined their attitude-behavior approach to different CSR initiatives such as environmental protection and philanthropic initiatives. The findings of this study have shown that there are differences between customer preferences and the environmental performance of banks.

So, the following research hypothesis is proposed:

- **Hypothesis 4 (H**_**4**_**):** The awareness of bank customers regarding the SDG practices of banks positively influences their satisfaction.

Customer trust in the banking sector eliminates potential consequences of financial crises (Järvinen 2014) and criticism of the sector for economic disparities and failures (Hurley et al. 2014). The strengthening of customer trust also improves the confidence on the financial decisions and products of the bank and secure that personal gains are not at stake, which is especially important for service sector companies. There is previous experience, which as a point can also act as a parabolic in case customers experience negative experiences from their banking institution. That is, there is a point at which when confidence levels are high, situations can be offset and counted as exceptions, which otherwise would act as a deterrent (van Esterik-Plasmeijer and Van Raaij 2017). Still, the trust notion is a special factor for the credibility of banks with its role intensifying after the financial crisis (Shim et al. 2013).

Trust is a focal point in the customer-banking business relationship; by publicizing their practices in sustainability initiatives, banks can create a more favorable environment. This favorable attitude may be transformed into corresponding customer behavior, in this case the factor of trust (Grayson et al. 2008). This suggests that sustainability practices can both build and further cultivate trust, which in turn will lead to more generally positive or improved customer perceptions of the business (Park et al. 2014). Based on the above, the following hypothesis is investigated:

- **Hypothesis 5 (H**_**5**_**):** The awareness of bank customers regarding SDG practices has a positive effect on trust.

The image of a bank is crucial in the decision of a potential customer to choose a bank. Thus, it is considered an important incentive to encourage banks to create a strong corporate image which could positively affect their customers’ behavior. Because of the positive effect of image on consumer behavior and generally on positive bias, image is positively associated with satisfaction (Keisidou et al. 2013; Tu and Chang 2012) and loyalty (Nguyen and Leblanc 2001; Stan et al. 2013). Sustainability management practices adopted by the banking sector seem to improve their image. By conducting a survey with 511 questionnaires about the banking sector, Igbudu et al. (2018) identified that the sustainable management of the sector positively affects customer loyalty and corporate image. Similarly, Özkan et al. (2020) shows that sustainability image and reputation affect customer loyalty. Therefore, the following hypotheses are investigated:

- **Hypothesis 6 (H**_**6**_**):** The better the sustainable image of banks as a result of SDGs, the greater the positive effect on customer loyalty.
- **Hypothesis 7 (H**_**7**_**):** The better the sustainable image of banks, the greater the positive effect on customer satisfaction.

Fair pricing policy relates to the customers’ psychological and financial understanding of the product or service they want. The perception of customers about fair pricing affects their satisfaction (Varki and Colgate 2001). Results of previous research in different industry sectors have confirmed that perceptions about the prices of products or services influence customer satisfaction (Fornell et al. 1996). The sustainability management of banks helps consumers to change their perception about fair pricing. The socio-environmental strategies of banks seem to be a key factor influencing customer perception of the way that financial products are priced and consequently customer confidence (Matute‐Vallejo et al. 2011). Thus, the following research hypothesis is proposed:

- **Hypothesis 8 (H**_**8**_**):** The better the perception of price fairness held by bank customers as a result of SDG practices, the greater the positive effect on their satisfaction.

Due to the importance they have on customer behavior, satisfaction and trust are two significant factors extensively studied in the literature,. The majority of current literature has shown a positive relationship between sustainability management, customer loyalty, and satisfaction (Amin et al. 2013; Hallowell 1996; Mosavi et al. 2018). Leninkumar (2017) identified that bank customer satisfaction is a very important factor which leads to customer loyalty.

- **Hypothesis 9 (H**_**9**_**):** Greater customer satisfaction as a result of SDGs also has a positive effect on their loyalty.

Satisfaction is the result of using a product or service, the feeling of pleasure or dissatisfaction derived from them. When this result has a positive balance, the emotional bonds with the organization are strengthened. Trust therefore increases as an already acquired trait from previous experiences with positive emotions (Leninkumar 2017; Mosavi et al. 2018). Moreover, although trust and satisfaction are based on experience, trust also contains the element of duration in a future expectation. Satisfaction, therefore, can be a factor influencing trust (Fassnacht and Köse 2007). Some studies have identified that CSR practices directly affect bank customers’ trust (Barcelos et al. 2015). Specifically, CSR and sustainability practices of the sector seem to have a critical role in customer satisfaction and trust. The following research hypothesis is examined:

- **Hypothesis 10 (H**_**10**_**):** The positive effect of SDGs on customer satisfaction also has a positive effect on their trust.

Empirical studies have proven the positive impact of trust on the loyalty of bank customers (Amin et al. 2013; Eisingerich and Bells 2007), with trust a precondition for its existence (Leninkumar 2017; Mosavi et al. 2018). Therefore, customers who have developed confidence in the services offered to them by an organization tend to redefine potential doubts and have a supportive attitude towards it (Omoregie et al. 2019). As previously mentioned, sustainability and CSR practices affect the trust and loyalty of banking customers. Realistically, the strengthening of customer trust as a result of SDGs could play a role in creating customer loyalty. Taking into account the preciously analyzed hypotheses:

- **Hypothesis 11 (H**_**11**_**):** The trust of bank customers as a result of SDGs also has a positive effect on their loyalty.

## Methodology

### Sample and procedure

A quantitative research design was developed to examine the above mentioned research hypotheses by means of a survey questionnaire method. The questionnaire consisted of structured questions related to the constructs in the proposed model and demographic characteristics of the respondents. Due to pandemic restrictions, the questionnaires were distributed online and collected from bank customers from April to June 2021 in Greece. The sample consisted of 1084 bank customers over 18 years old, 63% women and 37% men. The average age of the respondents was 37.5 years. Regarding the educational level, the largest percentage of the sample (43.7%) hold bachelor’s degrees, 27.4% a master’s, 4.2% PhDs, 24% are high school graduates, and the remaining percentage concerns primary school leavers.

To test the research hypothesis, the partial least squares approach to structural equation modeling was applied (PLS-SEM) which assists in maximizing the explanatory variability of endogenous structures (Hair et al. 2014). This technique has become a widely used multivariate analysis technique in examining consumer attitudes and behaviors. The SmartPLS 3.0 software was used to analyze the data.

### Measures and questionnaire structure

**Fig. 2:**
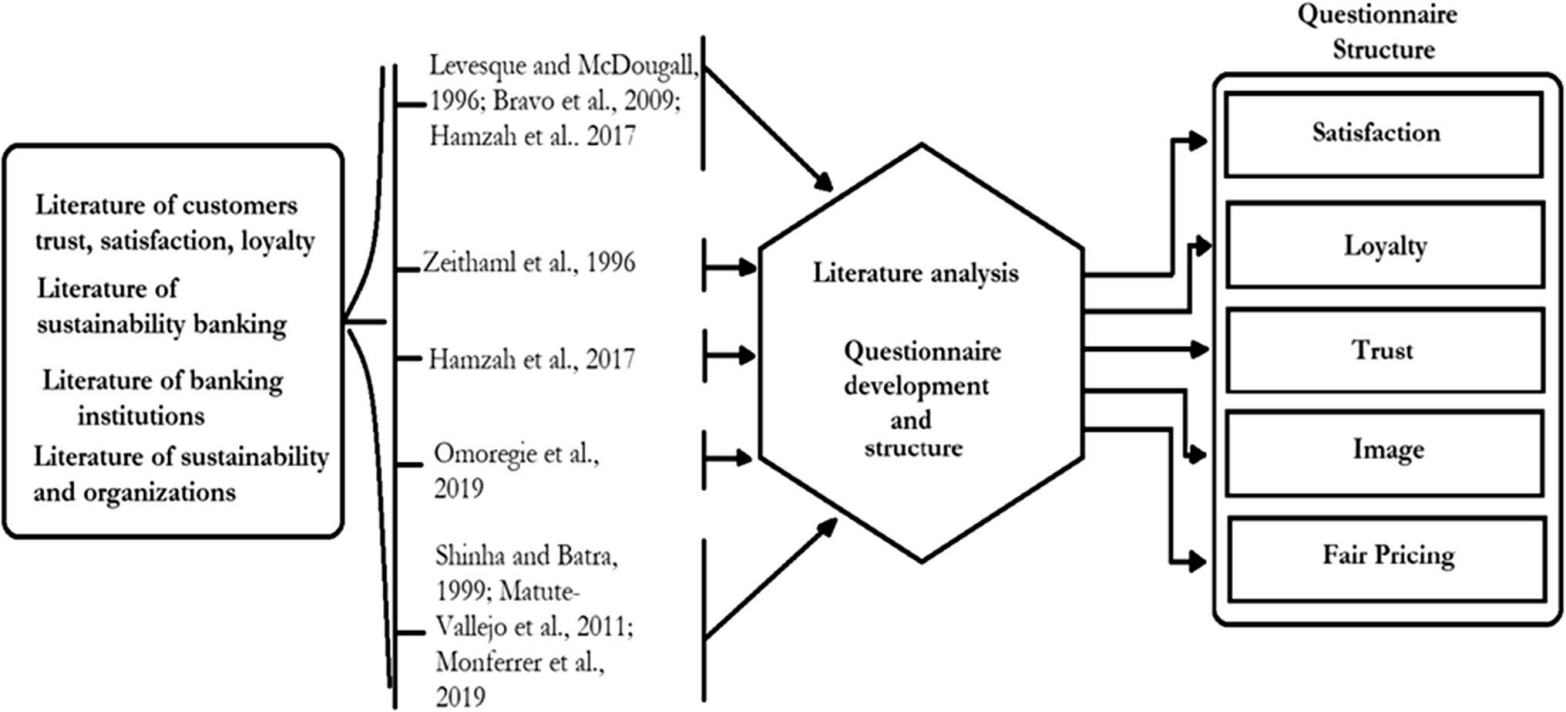
Questionnaire procedures and structure

The questionnaire has five sections to gather information to test hypotheses development as shown in Fig. 1. Additionally, Fig. 2 shows that the measurement data for was, sustainability banking, customer loyalty, trust, satisfaction, and sustainability of organizations. Firstly, the most relevant literature was gathered regarding each variable of this study including satisfaction, loyalty, trust, image, and fair pricing. Secondly, an analysis was conducted in order to identify the most significant and representative question for this study. Finally, a questionnaire was developed.

The SDG issues were identified from sustainability reports of banks. Ten sustainable practices in align with SDGs were identified and an assessment was carried out in order to examine if the customers are aware of them. To this end, a 5-point Likert scale from 1 (not at all) to 5 (very much) was utilized. Another 5-point Likert scale ranging from disagree (1) to strongly agree (5) was used to measure satisfaction, price fairness, image, and trust. The degree of probability (1 = very unlikely up to 5 = very probable) was used to measure loyalty. Finally, the degree of awareness of sustainable development goal practices was assessed on a scale from 1 (not at all) to 5 (very much).

## Results

### Measurement model

The PLS-SEM consists of the measurement model and the structural model. Table 1 presents the assessment of quality of measurement model. Loadings are above 0.708, which provides acceptable reliability, indicating that constructs explain more than 50% of the indicator’s variance (Hair et al. 2019). To assess internal consistency, reliability Cronbach’s *α* and composite reliability (CR) are used, meeting the criteria of values above 0.7 and indicating internal consistency. The metric for the evaluation of convergent validity is the average variance extracted (AVE) (Hair et al. 2017). AVE for all items on each construct above 0.5 indicate that the construct explains at least 50% of the variance of its items.

**Table 1:** Measurement model

Constructs	Items	Loadings	Cronbach’s *α*	CR	AVE
image	im1	0.898	0.860	0.914	0.781
	im2	0.900			
	im3	0.852			
loyal	lo1	0.917	0.922	0.941	0.763
	lo2	0.814			
	lo3	0.869			
	lo4	0.857			
	lo5	0.904			
pricf	prfr1	0.857	0.833	0.889	0.669
	prfr2	0.733			
	prfr3	0.836			
	prfr4	0.839			
satisf	satisf1	0.868	0.856	0.912	0.776
	satisf2	0.896			
	satisf3	0.879			
trust	tr1	0.929	0.818	0.916	0.846
	tr2	0.911			
sustpr	sustpr1	0.880	0.947	0.953	0.722
	sustpr2	0.829			
	sustpr3	0.858			
	sustpr4	0.774			
	sustpr5	0.890			
	sustpr6	0.796			
	sustpr7	0.848			
	sustpr8	0.868			
	sustpr9	0.872			
	sustpr10	0.878			

The Fornell-Larcker criterion and the heterotrait-monotrait ratio (HTMT) were used to evaluate discriminant validity (Table 2). The results follow the Fornell-Larcker criterion where for discriminant validity the square root of the AVE of any composite structure is higher than the correlation with any other composite structure (Fornell and Larcker 1981). Another measure for discriminant validity is the HTMT ratio. Henseler et al. (2015) proposed a value less than 0.85 between two constructs, which shows that discriminant validity is not present. The results present satisfactory of the measurement model.

**Table 2:** Fornell-Larcker criterion and heterotrait-monotrait ratio (HTMT)

	sustpr	image	loyal	pricf	satisf	trust
sustpr	*0.850*	0.381	0.371	0.388	0.312	0.407
image	0.356	*0.884*	0.712	0.581	0.724	0.801
loyal	0.356	0.635	*0.873*	0.596	0.763	0.685
pricf	0.351	0.493	0.523	*0.818*	0.610	0.685
satisf	0.290	0.625	0.680	0.518	*0.881*	0.769
trust	0.371	0.670	0.596	0.545	0.647	*0.920*

Below the diagonal are Fornell-Larcker criterion values and above the diagonal are HTMT values

### Structural model

The next step is the evaluation of PLS-SEM results. According to Hair et al. (2019), collinearity must be estimated to avoid issues of multicollinearity. Values of variance inflation factor (VIF) are below 0.5, showing no collinearity issue among the predictor constructs. Henseler et al. (2014) introduce the standardized root mean square residual (SRMR) in order to avoid model misspecification. The SRMR value of this research is 0.049, which shows that there is a good fit because it is lower than the threshold value of 0.08. A bootstrapping technique was performed for the examination of the model relationship hypotheses, the results of which are presented in Table 3.

**Table 3:** Path coefficient, *β*, *t*-values of the structural model

Hypothesis	*β*	*Τ*	Result
Η_1_: sustpr → image	0.356	13.752	Supported
Η_2_: sustpr → loyal	0.099	4.744	Supported
Η_3_: sustpr → pricf	0.351	12.932	Supported
Η_4_: sustpr → satisf	0.023	0.854	Not supported
Η_5_: sustpr → trust	0.200	8.886	Supported
Η_6_: image → loyal	0.266	8.901	Supported
Η_7_: image → satisf	0.483	14.323	Supported
Η_8_: pricf → satisf	0.272	8.835	Supported
Η_9_: satisf → loyal	0.411	13.163	Supported
Η_10_: satisf → trust	0.589	26.075	Supported
Η_11_: trust → loyal	0.115	3.797	Supported

Results show that all relationships are positive and statistically significant (*p* < 0.001) except H_4_ (sustpr → satisf: *β* = 0.023, *Τ* = 0.854). Awareness of SDG practices has no significant effect on customer satisfaction; thus unexpectedly, H_4_ is not supported. Awareness of SDG practices has a significantly positive effect on image (sustpr → image: *β* = 0.356, *Τ* = 13.752), loyalty (sustpr → loyal: *β* = 0.099, *Τ* = 4.744), perceived fair pricing policy (sustpr → pricf: *β* = 0.351, *Τ* = 12.392), and trust (sustpr → trust: *β* = 0.200, *Τ* = 8.886). Thus, H_1_, H_2_, H_3_, and H_5_ are supported. Furthermore, image has an effect on customer loyalty (image → loyal: *β* = 0.226, *Τ* = 8.901) and satisfaction (image → satisf: *β* = 0.483, *Τ* = 14.323). Hence, H_6_ and H_7_ are supported. Price fairness as a result of SDG awareness has a significant positive effect on bank customer satisfaction (price fair → satisfaction: *β* = 0.272, *Τ* = 8.835), which supports Η_8_. Additionally, satisfaction as an effect of SDGs has an impact on loyalty (satisf → loyal: *β* = 0.411, *Τ* = 13.163) and trust (satisf → trust: *β* = 0.589, *Τ* = 26.075); thus, hypothesis H_9_ and H_10_ are supported. Finally, trust as an effect of SDGs has a significant effect on loyalty (trust → loyal: *β* = 0.115, *Τ* = 3.797), so Η_11_ is supported. The results showed moderate explanatory power for image, price fairness, satisfaction, and trust (*R*^2^ are 0.127, 0.123, 0.449, and 0.455, respectively) and substantial loyalty (*R*^2^ = 0.552). Additionally, *Q*^2^ values of image (0.098), loyalty (0.417), price fairness (0.081), satisfaction (0.343), and trust (0.381) are above zero, which indicates predictive accuracy. The findings show that the suggested model provides significant insights regarding the effects of SDGs on bank customers’ behavior.

## Discussion

The paper contributes to current literature in a theoretical and practical manner. Firstly, it contributes by examining the limited banking sustainability literature through an SDGs lens. So far, the majority of current literature has mainly focused on analyzing how banks meet the SDGs through sustainability reporting evaluation. It contributes by showing how banks have extended sustainability strategies from triple-bottom-line to a broad spectrum of 17 SDGs. The real value of it is that it overcomes the current normative models which suggest how banking sector addresses SDGs and assesses what banks do in reality to meet SDGs.

Another significant contribution of this research lies in the fact that the awareness of SDGs by customers becomes an important factor in their strategy. The findings have highlighted that the most significant factor for customers is the effect of SDG practices adopted by banks. This implies that the more customers know about the SDG practices of banks and their sustainability image, the more it would influence potential customers to become actual customers. Indeed, information on SDGs enhances the image of banks. This finding is in line with previous studies about the relationship of banking sustainability management and CSR strategies on their image. By examining 213 Iranian bank customers, Salehzadeh et al. (2018) identified the influence of CSR strategies on the image of banks and the awareness of customers. Pérez and del Bosque (2015c) have identified the association between consumer preferences and the CSR image of banking institutions. This paper also contributes to previous surveys by broadening through the SDGs perspective the narrow content of analysis of the role of corporate social responsible initiatives and the company-related associations of stakeholders (Bhattacharya et al. 2009).

An additional influence of this paper is to examine the awareness of bank customers regarding the role of SDGs adopted by banks in their perception about fair pricing policy of banks. The findings show that banks that share their performance regarding SDGs increase customer awareness about their fair pricing policy. One rational explanation of this finding is that a responsible institution follows similar practices for all their products, services, and operations in relation to customers and stakeholders. This study broadens the narrow focus of previous studies which were only on environmental responsibility by taking the broader perspective of sustainability goals (Matute-Vallejo et al. 2011). The way that banking institutions use the SDGs to create a better image should take into account stakeholders’ needs in order to improve customer confidence and create value for the banking sector (Yamane and Kaneko 2021).

This paper also contributes by examining how trust of banking customers is related to SDGs integration. It focuses on the idea that trust is another significant feature that seems to be influenced by the banks’ SDGs. The findings show that the negative or positive view of bank customers about SDGs could affect their trust in relation to banks. Bank customers who are aware of practices related to SDGs have greater confidence in their bank. This is particularly important for the sector, which is often plagued by a lack of trust due to the nature of its activity but also due to social habits and economic crises. So, it is interesting how awareness of banks’ contribution to sustainable development goals can strengthen trust. Information should be properly communicated and based on real actions so as not to test trust. It has been shown that, in the case of negative information, the negative effect will be evident in consumer attitudes, who are more sensitive to negative information than in the positive one (Sen and Bhattacharya 2001). The findings are in line with previous studies of the CSR and sustainability literature for the banking sector (Bugandwa et al. 2021). They identified that some strategies of CSR play a critical role in the customer trust such as legal compliance, social norms, product responsibility, environmental protection, and employee relations.

The effect of SDGs of the sector on customer loyalty is another contribution of this paper. Loyalty is a critical factor that influences customer behavior, exceeding feelings of satisfaction and trust; the customer makes an emotional bond with the bank and creates an emotional loyalty (Oliver 1999). This is also a key point, because it has been emphasized that usually there is lack of emotional commitment between banks and their customers (De Chernatony and Riley 1999). Findings of this paper show that customer loyalty is therefore positively influenced by the SDGs of banks. Although this type of loyalty is not very significant, is also valuable due to the fact that in many studies, the direct effect on loyalty is not considered (Han et al. 2018; Park and Kim 2019). It is important to point out that this paper offers an alternative approach to examine the direct effect of customer awareness about the banking sector’s SDGs on their loyalty and it transpired that there is a relationship between the two structures. Therefore, these findings complement previous studies (Sen et al. 2006). Focusing on CSR initiatives and purchasing behavior, it appears that awareness of sustainable development goals can strengthen customers’ emotional commitment to their banking institution.

In addition, it should be noted that the impact of the sector’s SDGs on customers’ satisfaction has not been confirmed. Previous research confirms the effect of the sector’s CSR practices on customer satisfaction (Chung et al. 2015; Luo and Bhattacharya 2006; Paulík et al. 2015). Regarding this relationship, it has been argued that the efforts of banks to invest in CSR initiatives may not have significant returns (McDonald and Rundle-Thiele 2008). Although satisfaction is examined both in marketing and consumer studies, this paper examines awareness of SDG practices which constitute a broader and more comprehensive framework for achieving sustainability. Findings indicate no relationship for the impact of sustainable awareness practices in customer satisfaction. One reason to explain this relationship could be that customer sustainability awareness creates positive attitudes and behaviors in the other factors considered. This means that customers support these efforts in order to achieve sustainability. Specifically, customers respect their bank for incorporating SDGs in their design and support this implementation. This is already proven by the positive effect of SDGs on factors such as the bank image, trust, perceived fair pricing policy, and loyalty.

However, it should highlight that supporting SDG practices does not necessarily mean that customers are satisfied with the use of the products and services offered. Customers might support SDG practices in the case where they realize the importance of the contribution and not necessarily when they get personal gains. Overall, findings showed that sustainability awareness has a positive effect on the behavior and intentions of bank customers and constitutes an exploitable path from decision makers to strengthen the behavioral structures of bank customers.

## Conclusions

This paper examines the impact of bank customers’ awareness of SDG practices on their behavioral intentions and attitudes to bank products. The aim was to analyze how and whether banks’ strategies to achieve SDGs influence customers’ behaviors related to the perceived image, fair pricing policy, trust, satisfaction, and loyalty. In particular, the awareness of all objectives as a single factor was investigated in order to examine whether the holistic approach to sustainability has an impact on the way customers of banking institutions react and behave.

Overall, the results highlighted the positive attitude of customers to these behaviors. Sustainable awareness has an impact on all the factors, except for satisfaction where the relationship was not found to be significant. As it is the case for all organizations, customers are an important group of stakeholders for the banking sector and their personal beliefs and values are often incorporated into their consumer preferences. When customers get information regarding banks’ SDGs, they show a more positive attitude towards the bank and this in turn can influence businesses to be more sustainable.

Although this study explains the integration of SDG practices in relation to consumer behavior, there are some limitations. These limitations provide opportunities for future research and further exploration of the subject. One significant limitation concerns the geographical focus of this study on only one area. The research was conducted on customers of Greek banks living in Greece. This focus is very interesting to understand how bank customers behave within the same institutional context, with the same habits and beliefs. However, it would be of great interest to investigate the impact of awareness of SDG practices in other countries with different economic and social backgrounds. In this case, a comparison could be made if information has the same effects on the behavior of bank customers. Another significant limitation is that the findings of this study do not allow the generalization of results arising from the specific sector. This work highlights the role of sustainability awareness at an early stage in customer behavior. So, it is proposed to examine these impacts in different sectors in order to examine whether and how strong the influence of sustainability awareness on consumer behavior is to other products or services.

## Data Availability

All data used in this research are available for check from the corresponding author on request of the Editor.

## Appendix

**Table:**

A 1. Items	
sustpr1	It offers training programs for its staff but also in general education of the citizens through collaborations and sponsorships
sustpr2	Ensures the protection of personal data and their processing in a legal manner. It takes action against cyber fraud while informing its customers accordingly
sustpr3	Introduces environmental criteria in business loans
sustpr4	It offers the same development opportunities to male and female employees
sustpr5	Utilizes new technological solutions to upgrade the products and services provided for the benefit of its customers
sustpr6	Offers the same employment opportunities to people with disabilities, different sexual orientation, nationality, religion, and culture
sustpr7	Emphasizes customer service, aiming at transparency in the communication of its products and services. There is the possibility for customers to submit complaints to the bank in a clear and understandable way
sustpr8	Supports companies with innovative ideas and high growth capabilities (for example, start up)
sustpr9	Helps reduce paper and ink usage by selecting online updates over paperback
sustpr10	Supports programs—investments in renewable energy source
